# Tachykinin-Related Peptides Share a G Protein-Coupled Receptor with Ion Transport Peptide-Like in the Silkworm *Bombyx mori*

**DOI:** 10.1371/journal.pone.0156501

**Published:** 2016-06-01

**Authors:** Chiaki Nagai-Okatani, Hiromichi Nagasawa, Shinji Nagata

**Affiliations:** 1 Department of Applied Biological Chemistry, Graduate School of Agricultural and Life Sciences, The University of Tokyo, Tokyo, Japan; 2 Department of Integrated Biosciences, Graduate School of Frontier Sciences, The University of Tokyo, Chiba, Japan; Wake Forest University, UNITED STATES

## Abstract

Recently, we identified an orphan *Bombyx mori* neuropeptide G protein-coupled receptor (BNGR)-A24 as an ion transport peptide-like (ITPL) receptor. BNGR-A24 belongs to the same clade as BNGR-A32 and -A33, which were recently identified as natalisin receptors. Since these three BNGRs share high similarities with known receptors for tachykinin-related peptides (TRPs), we examined whether these BNGRs can function as physiological receptors for five endogenous *B*. *mori* TRPs (TK-1–5). In a heterologous expression system, BNGR-A24 acted as a receptor for all five TRPs. In contrast, BNGR-A32 responded only to TK-5, and BNGR-A33 did not respond to any of the TRPs. These findings are consistent with recent studies on the ligand preferences for *B*. *mori* natalisins. Furthermore, we evaluated whether the binding of ITPL and TRPs to BNGR-A24 is competitive by using a Ca^2+^ imaging assay. Concomitant addition of a TRP receptor antagonist, spantide I, reduced the responses of BNGR-A24 not only to TK-4 but also to ITPL. The results of a binding assay using fluorescent-labeled BNGR-A24 and ligands demonstrated that the binding of ITPL to BNGR-A24 was inhibited by TK-4 as well as by spantide I, and *vice versa*. In addition, the ITPL-induced increase in cGMP levels of BNGR-A24-expressing BmN cells was suppressed by the addition of excess TK-4 or spantide I. The intracellular levels of cAMP and cGMP, as second messenger candidates of the TRP signaling, were not altered by the five TRPs, suggesting that these peptides act *via* different signaling pathways from cAMP and cGMP signaling at least in BmN cells. Taken together, the present findings suggest that ITPL and TRPs are endogenous orthosteric ligands of BNGR-A24 that may activate discrete signaling pathways. This receptor, which shares orthosteric ligands, may constitute an important model for studying ligand-biased signaling.

## Introduction

Ion transport peptide (ITP) and its alternatively spliced variant, ITP-like (ITPL), are insect peptides that belong to the crustacean hyperglycemic hormone (CHH) superfamily [1–4]. These neuropeptides have been identified in many ecdysozoans, including insects, crustaceans, and nematodes. Members of this neuropeptide family are composed of 70–80 amino acid residues, showing structural similarity with three conserved intramolecular disulfide bonds. The insect members of this family, ITP and ITPL, were originally identified as regulators of ion and fluid transport across the locust ileum [5,6]. ITP and ITPL are differently expressed in various tissues, indicating they have extensive and discrete functions. In *Drosophila*, ITP functions in the regulation of the circadian rhythm [7–9] and has been suggested to be involved in ecdysis in the tobacco hornworm *Manduca sexta* [10]. ITPL plays a role in the ovarian maturation of the red flour beetle *Tribolium castaneum* [11]. However, the functions of ITP [12] and ITPL [13] in the silkworm *Bombyx mori* remain unclear.

Recently, by screening for orphan neuropeptide G protein-coupled receptors (GPCRs) in *B*. *mori* (BNGRs) [14], we successfully identified three BNGRs (BNGR-A2, -A24, and -A34) as functional receptors for endogenous ITP and ITPL [15]. The two ITP receptors, BNGR-A2 and -A34, and the ITPL receptor, BNGR-A24, belong to the class A (rhodopsin-like) GPCR family. While the two ITP receptors are newly deorphanized, BNGR-A24 appears to shares high similarity with tachykinin receptors.

Tachykinins and their structurally related peptides, comprise a large family of multifunctional brain-gut peptides found in both vertebrates and invertebrates [16–20]. One of the most well-known tachykinin family members is substance P. In invertebrates, tachykinin family members can be structurally divided into two types: invertebrate tachykinins (invTKs) containing a vertebrate-like C-terminal F*X*GLMa (‘a’ indicates C-terminal amidation); and tachykinin-related peptides (TRPs) with a C-terminal F*X*G/A*X*Ra (Gly for the third residue appears more frequently) motif. TRPs are considered as functional counterparts of vertebrate tachykinins and are expressed in the nervous system, gastrointestinal tract, and many other tissues. An extensive range of biological activities, including modulation of muscle contraction, diuretic activity, neuromodulatory effects, and induction of adipokinetic hormone secretion, has been observed in insects using *in vitro* bioassays [19].

In *B*. *mori*, five endogenous TRPs are produced from a single precursor polypeptide encoded by the tachykinin gene [21]. Peptidomic analysis has demonstrated the presence of these TRPs in *B*. *mori* larvae, pupae, and adults [22]. To date, the physiological roles of TRPs have been demonstrated in the induction of pheromone biosynthesis [23] and the modulation of feeding behavior [24]. However, little is known regarding the biological functions of TRPs in *B*. *mori*.

Although several bioinformatic studies have identified the structure and presence of TRPs in various invertebrates, the functional characterization of TRP receptors has not been performed extensively thus far. *In silico* comparative analyses of GPCRs in different insect species have suggested that BNGR-A24, -A32, and -A33 are candidate TRP receptors in *B*. *mori* [14,15,25]. BNGR-A24 has been shown to respond to one TRP in *D*. *melanogaster*, DTK-6 [26] and BNGR-A32 and -A33 have been identified recently as receptors for endogenous natalisins, which function in reproduction and contain the TRP-like C-terminal motif (F/Y*XXX*Ra) [26]. To date, it remains unclear whether BNGR-A24, -A32, and -A33 function as receptors for the five endogenous TRPs in *B*. *mori*.

In the present study, we determined whether candidate BNGRs function as receptors for *B*. *mori* TRPs using a Ca^2+^ imaging technique. In addition, we evaluated the possibility of competitive binding *in vivo*, since BNGR-A24 functions as a physiological receptor for both TRP and ITPL.

## Materials and Methods

### Insects

Silkworm eggs of the *B*. *mori* racial hybrid, Kinshu × Showa, were purchased from Ueda Sanshu (Nagano, Japan) and used for all experiments. Larvae were reared in plastic containers at 26 ± 1°C with 70 ± 10% relative humidity under long-day lighting conditions (16 h light/ 8 h dark) and fed with SILKMATE 2S artificial diet (Nihon Nosan Kogyo, Yokohama, Japan).

### Preparation of recombinant and synthetic peptides

Recombinant *B*. *mori* ITPL (rITPL) was prepared as previously reported [15]. rITPL was collected as inclusion bodies from an *E*. *coli* expression system and the inclusion body components were denatured and refolded. Purification of rITPL was performed by reversed-phase high performance liquid chromatography (HPLC) on a Shodex Asahipak ODP-50 column (10 mm inner diameter × 250 mm, Showa Denko, Tokyo, Japan), using a 30-min linear gradient of 20–50% acetonitrile, 0.05% trifluoroacetic acid, at a flow rate of 3 mL/min. Elution was monitored by absorbance at 280 nm. *B*. *mori* TRPs were chemically synthesized on an automated Apex 369 peptide synthesizer (AAPPTec, Louisville, KY, USA), using a standard Fmoc (*N*-[9-fluorenylmethoxycarbonyl]) solid-phase protocol and reagents purchased from Watanabe Chemical Industries (Hiroshima, Japan). Following deprotection and cleavage from the resin, the synthetic TRPs were purified by reversed-phase HPLC on a PEGASIL-300 ODS column (10 mm inner diameter × 250 mm, Senshu Kagaku, Tokyo, Japan), using a 30-min linear gradient of 10–40% acetonitrile, 0.05% trifluoroacetic acid, at a flow rate of 1 mL/min. Elution was monitored by absorbance at 225 nm. Synthetic spantide I was purchased from Peptide Institute (Osaka, Japan). Fluorescent-labeled peptides were prepared using rhodamine red (RR) succinimidyl ester (Life Technologies, Tokyo, Japan), according to manufacturer’s protocols, and were purified by reversed-phase HPLC under the same conditions as the TRPs.

### Expression analysis by RT-PCR

Tissues were dissected in 0.9% NaCl solution from anesthetized *B*. *mori* larvae (three males and three females) at fifth instar day 2 that were fed *ad libitum* or starved for 24 h. Total RNA was extracted from the tissues using TRIzol Reagent (Life Technologies). cDNA synthesis was performed with SuperScript III (Life Technologies) and an oligo (dT)_12-18_ primer. Partial cDNA fragments of the target genes were amplified with Go Taq DNA polymerase (Promega, Tokyo, Japan) and the following primers: 5′-ATCAACGACGGCCAATACCC-3′ and 5′-TCCCGAAGAATCCCATCTGC-3′ for the tachykinin gene (*tk*, GenBank accession number NM_001130892); and 5′-GGTTCACTGTCGCGTTTCTA-3′ and 5′-TCTTTCCACGATCAGCTTCC-3′ for the ribosomal protein L32 gene (*rpl32*, GenBank accession number NM_001098282). The amplification conditions were as follows: 20 s (140 s for the first cycle) at 94°C, 30 s at 60°C, and 45 s at 72°C for 30 cycles (for *tk*) or 20 cycles (for *rpl32*). Densitometric analysis was performed for three independent experiments using the ImageJ software from the National Institutes of Health (http://imagej.nih.gov/ij/).

### Ca^2+^ imaging assay of BNGRs

The assays were performed as previously reported [15]. Briefly, HEK293T cells seeded on a glass-based dish were co-transfected with pME18S plasmids carrying BNGRs and the promiscuous G protein alpha subunit, Gα15. Following 24 h incubation, the transfected cells were loaded with 500 μL of a Ca^2+^ indicator solution (2 μM Fluo-4 AM [Dojin Chemicals, Kumamoto, Japan] and 0.01% Pluronic-F 127 [Anaspec, San Jose, CA, USA] in Opti-MEM [Life Technologies]) and incubated at 37°C for 30 min in a dark room. The cells were washed twice with Ringer’s solution and then 800 μL Ringer’s solution was added to the cells. Within a given run time, 100 μL of the following compounds were added sequentially: Ringer’s solution (at 15 s, vehicle control), the peptide of interest or a mixture with its potential competitor (at 60 s), and 100 μM ATP (at 120 s, an experimental control). Fluo-4-derived fluorescence was chronologically monitored using a confocal laser-scanning microscope (FV-1000D; Olympus, Tokyo, Japan), with the preinstalled dye mode for Fluo-3. At least three independent experiments were performed. Dose-response curves were generated and EC_50_ values were calculated from the obtained fitting curves.

### Competitive binding assay

Binding assays were performed as previously reported [15]. Briefly, CHO cells were transfected with 2 μg of pME18S plasmids harboring enhanced green fluorescent protein (EGFP)-fused BNGR cDNA. The binding assay was performed with cells 24 h post transfection. The cells were pre-incubated at 4°C for 30 min prior to the assay to minimize receptor internalization by ligand binding. Following the addition of 50 μL of non-labeled ligand as a potential competitor, 50 μL of RR-labeled ligand was added. Subsequent to incubation at 4°C for 60 min, the cells were washed twice with an ice-cold fixation buffer (4% formaldehyde in PBS) and then 500 μL of the fixation buffer was added. The fluorescence derived from EGFP and RR was observed using a confocal microscope (FV-1000D) with preinstalled dye modes for EGFP and DsRed, respectively. The fluorescent data obtained from six individual cells of at least two independent experiments were analyzed using Fluoview software (Olympus).

### cAMP assay and competitive cGMP assay

BmN cells were plated in 12-well tissue culture plates (2 × 10^5^ cells/well; Asahi glass, Yokohama, Japan) and maintained in normal growth medium (TC-100 [Applichem, Darmstadt, Germany], 10% FBS, and 50 μg/mL gentamycin). At 3 days post-seeding, the cells were pre-incubated in 800 μL of assay medium (TC-100 supplemented with 1 mM 1-methyl-3-isobutylxanthine [IBMX; Sigma-Aldrich, Tokyo, Japan]) and 1 mM phenylmethylsulfonyl fluoride for 10 min at 28°C. Following pre-incubation, 100 μL of a potential competitor and 100 μL of the peptide of interest were added sequentially to the medium and incubated at 28°C for 30 min. After incubation, the cells were washed twice with TC-100 and then immediately frozen in liquid nitrogen. Intracellular cAMP and cGMP were extracted by sonication with 300 μL 0.1 M HCl supplemented with 1 mM IBMX and then collected from the supernatant subsequent to centrifugation (4°C, 16,000 × *g*, 10 min). The extracted cAMP and cGMP were quantified in duplicate using a cAMP ELISA kit (Cayman Chemicals, Ann Arbor, MI, USA) and a cGMP EIA system (GE Healthcare, Hino, Japan), respectively. These experiments were repeated at least twice.

## Results

### Tissue distribution of the tachykinin gene

**Fig 1:**
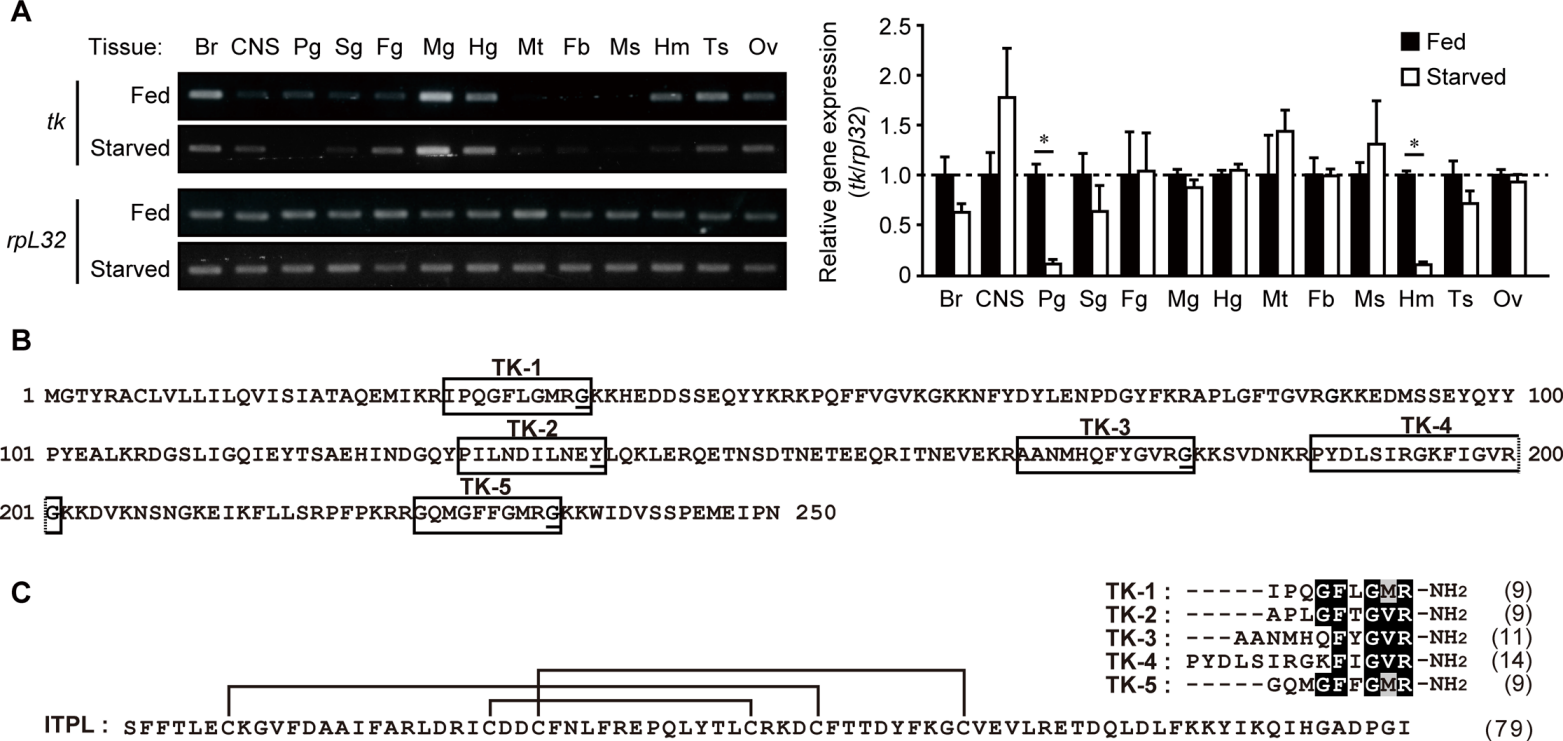
Endogenous TRPs in *B*. *mori*. (A) Tissue distribution and expression profile of the tachykinin gene (*tk*) in *B*. *mori* larvae. Tissues from six individual fifth-instar day 2 larvae fed *ad libitum* or starved for 24 h were used for this semi-quantitative RT-PCR analysis. Ribosomal protein L32 gene (*rpl32*) was used as an experimental and expression control. The left panel is a representative image of the analyses. The right panel shows the densitometric analysis results from three independent experiments. The starved larvae data are depicted as the fold change over that in the same tissue of fed larvae (mean + S.D.). Asterisks indicate significant differences between the two groups for the same tissue (Fed vs. Starved; *P* < 0.05, unpaired *t*-test). *Br*, brain; *CNS*, central nervous system; *Pg*, prothoracic glands; *Sg*, silk gland; *Fg*, foregut; *Mg*, midgut; *Hg*, hindgut; *Mt*, Malpighian tubules; *Fb*, fat body; *Ms*, smooth muscle from the 8th body segment; *Hm*, hemocytes; *Ts*, testis; *Ov*, ovary. (B) Amino acid sequence of the polypeptide encoded by the tachykinin gene; this precursor is processed to produce five premature peptides (boxed), which are subsequently amidated with the C-terminal Gly residue serving as a donor (underlined) that requires maturation. (C) Sequence comparison of the five mature TRPs and ITPL present in *B*. *mori*. ITPL contains three intramolecular disulfide bonds (solid lines). The amino acid sequences of the five TRPs were aligned using ClustalW (http://clustalw.ddbj.nig.ac.jp); these peptides contain the consensus sequence (-Phe-X_1_-Gly-X_2_-Arg-NH_2_) of TRPs found in invertebrates. Numbers in parentheses indicate the number of amino acid residues.

Previously, we showed that *bngr-A24* is ubiquitously expressed in the tissues of fifth instar day 2 larvae and that gene expression of both *bngr-A24* and *itpl* in the intestinal tissues is upregulated by 24 h starvation [15]. In order to determine in which tissues the tachykinin gene, *tk*, is expressed and whether *tk* expression is affected by feeding state, we analyzed the expression profile of *tk* by RT-PCR. In larvae fed *ad libitum*, *tk* expression was relatively higher in the midgut; moderate in the brain, hindgut, hemocytes, and reproductive tissues; and relatively lower in the central nervous system, prothoracic glands, silk gland, and foregut. Very low levels of PCR product were detected in the Malpighian tubules, fat body, or smooth muscle (Fig 1A, left panel, Fed). The *tk* expression profile in larvae following 24 h starvation was similar to that of larvae fed *ad libitum* (Fig 1A, left panel, Starved), except for a notable reduction of expression in the prothoracic glands and hemocytes (Fig 1A, right panel).

### Response of candidate BNGRs to *B*. *mori* TRPs

**Fig 2:**
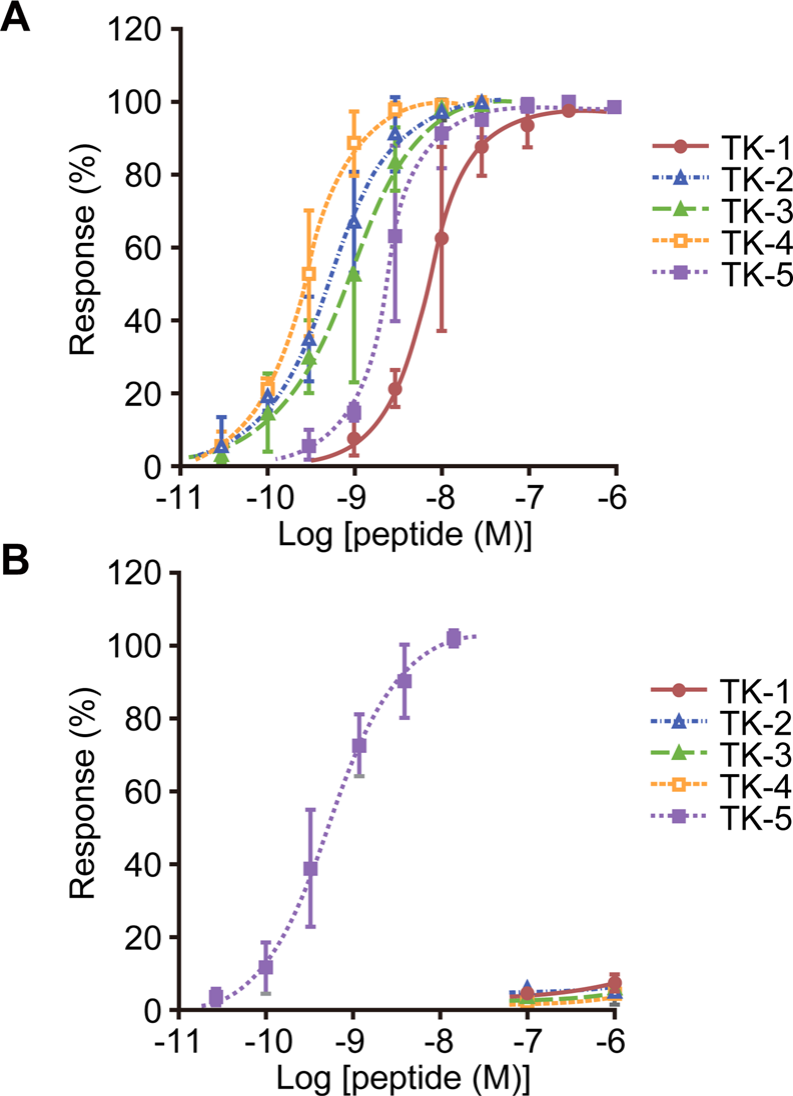
Responses of BNGR-A24 and BNGR-A32 to *B*. *mori* TRPs. The response of HEK293T cells co-expressing BNGR-A24 (A) or BNGR-A32 (B) and promiscuous mouse Gα15 to the TRPs was monitored using the Ca^2+^ imaging technique. Dose-response curves are depicted as relative fluorescent intensity (mean ± S.D.; *n* = 3).

The *B*. *mori tk* gene encodes a 250-amino acid polypeptide (Fig 1B), which is a precursor of five TRPs, TK-1–5 (Fig 1C). In order to evaluate whether the three TRP receptor candidates, BNGR-A24, -A32, and -A33, function as receptors for *B*. *mori* TRPs, the response of these three BNGRs to chemically synthesized TRPs was examined using the Ca^2+^ imaging assay. When BNGR-A24 was heterologously expressed in HEK293T cells, it showed dose-dependent response to all five TRPs; the maximum response was observed against TK-4, with an EC_50_ = 0.27 nM (Fig 2A and Table 1). In addition, BNGR-A32 responded to TK-5 with an EC_50_ = 0.48 nM; however, almost no response was observed for TK-1–4 at concentrations as high as 1 μM (Fig 2B and Table 1). In contrast, BNGR-A33 did not exhibit an obvious response to a 1 μM concentration of any of the five TRPs. These results indicate that BNGR-A24 and -A32, but not BNGR-A33, function as receptors for endogenous TRPs in *B*. *mori*.

**Table 1:** EC_50_ of BNGR responses to *B*. *mori* TRPs.

Receptor	Ligand	EC_50_ (nM)^*a*^
BNGR-A24	TK-1	7.1
	TK-2	0.49
	TK-3	0.79
	TK-4	0.27
	TK-5	2.3
BNGR-A32	TK-5	0.48

^a^EC_50_ values were calculated using the Ca^2+^ imaging assays shown in Fig 2.

The EC_50_ values of BNGR-A32 to TK-1–4 and BNGR-A33 to TK-1–5 were not calculated because of their lower response levels.

### Evaluation of the competitive binding of ITPL and TRPs to BNGR-A24

**Fig 3:**
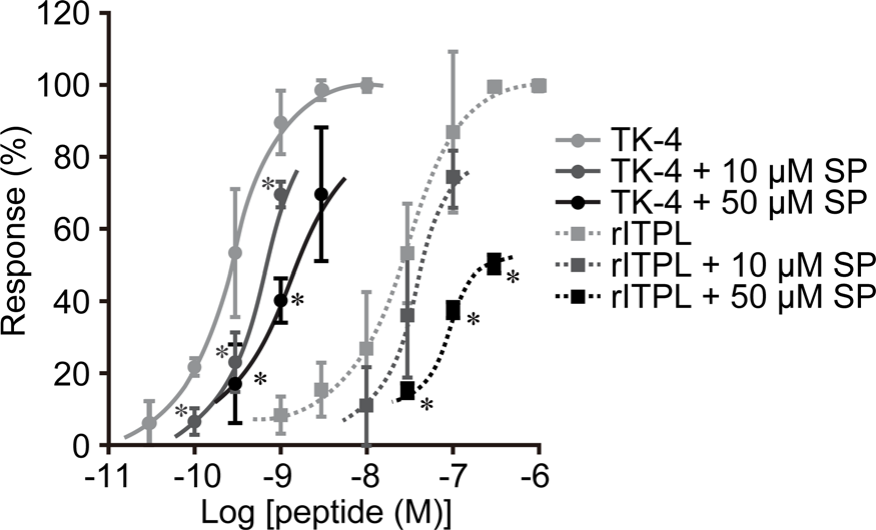
Effect of spantide I on the response of BNGR-A24 to rITPL and TK-4. Spantide I (*SP*), a potential competitor, was added together with rITPL or TK-4 and the response of BNGR-A24 was monitored using the Ca^2+^ imaging assay. Dose-response curves of the responses are presented as relative fluorescent intensity (mean ± S.D.; *n* = 3). The response curve of TK-4 with no competitor is the same as the one shown in Fig 2A. Asterisks indicate significant differences for treatment with the same ligand without spantide I (*P* < 0.05; Dunnett’s test).

The results shown in Fig 2A and Table 1 and our previous report [15] indicate that BNGR-A24 is a functional receptor not only for ITPL but also for the five TRPs, suggesting the possibility of competitive binding of ITPL and the TRPs to BNGR-A24. To address this question, we examined whether the responses of BNGR-A24 to rITPL and the TRPs are affected by a potential competitor, spantide I, which is an antagonistic analog of substance P. Simultaneous treatment of BNGR-A24 with 10 μM or 50 μM of spantide I significantly decreased its response to TK-4 (Fig 3). The response of BNGR-A24 to TK-4 was successively decreased in a dose-dependent manner with increasing concentration of spantide I. These results indicate that spantide I functions as a TRP antagonist for BNGR-A24. Next, we examined the effect of simultaneous addition of spantide I and rITPL on ligand competition. The dose-response curve of 10–300 nM rITPL shifted in a similar manner to that observed for TK-4. Furthermore, when 50 μM of spantide I was added, the response of BNGR-A24 to rITPL was significantly lower than that without the competitor. These results indicate that ITPL binding to BNGR-A24 was inhibited by spantide I, a competitive antagonist of both tachykinins and TRPs.

**Fig 4:**
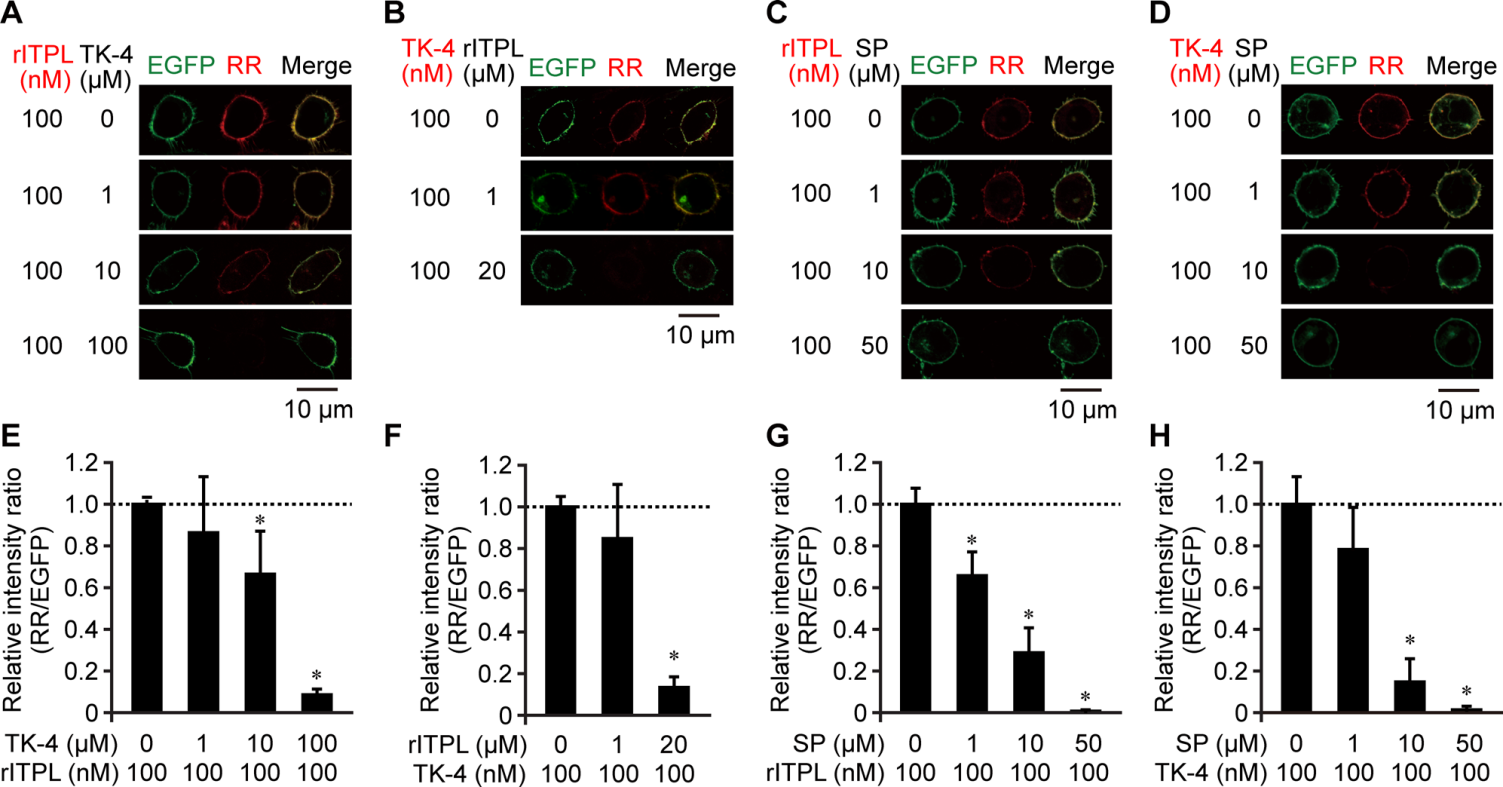
*In vitro* competitive binding assay of rITPL and TK-4 to BNGR-A24. (A–D) Microscopic imaging. The binding of RR-labeled ligands (A and C, rITPL; B and D, TK-4) and CHO cells expressing EGFP-fused BNGR-A24 was examined in the presence of potential competitors (A, TK-4; B, rITPL; C and D, spantide I [SP]). EGFP and RR fluorescences were observed by confocal microscopy. Co-localization is presented as yellow in the merged images (Merge). Representative images of cells from at least two independent experiments are shown. (E–H) Relative fluorescent intensity obtained from experiment images (A)–(D), respectively. RR fluorescent intensity was normalized by EGFP intensity per image and is indicated as fold change over that without competitor (mean + S.D.; *n* = 6). Asterisks indicate significant differences compared to ‘no competitor’ data (*P* < 0.05, Dunnett’s test).

In order to directly demonstrate the competitive binding of ITPL and TRPs to BNGR-A24, we performed an *in vitro* binding assay using RR-labeled ligands and CHO cells expressing BNGR-A24. Following 30-min exposure of the cells to RR-labeled rITPL or TK-4, both red fluorescence (derived from the RR-labeled ligands) and green fluorescence (derived from EGFP C-terminally fused to BNGR-A24) were observed on the cell surface, indicating ligand-receptor binding (Fig 4). When 1–100 μM of unlabeled TK-4 was added prior to the addition of RR-labeled rITPL, the fluorescence of labeled rITPL on the cell surface gradually decreased with increasing TK-4 dose (Fig 4A and 4E). A similar result was obtained using RR-labeled TK-4 and unlabeled rITPL (Fig 4B and 4F). In this case, the addition of 20 μM rITPL reduced the ligand-derived signal by approximately 90% compared to that observed without the addition of unlabeled ligand. To confirm the results obtained from the Ca^2+^ imaging assay, we examined binding in the presence of spantide I, a competitor of rITPL and TK-4; our results show that the fluorescence intensity of the RR-labeled ligands decreased in response to increasing doses of spantide I (Fig 4C, 4D, 4G and 4H). Addition of 50 μM spantide I was sufficient for nearly complete quenching of the fluorescence derived from RR-labeled rITPL and TK-4. This competitive effect of spantide I against rITPL suggests that other TRPs in addition to TK-4 act as competitors against ITPL binding to BNGR-A24.

### Effect of ITPL and TRP competition for BNGR-A24 on signal transduction

**Fig 5:**
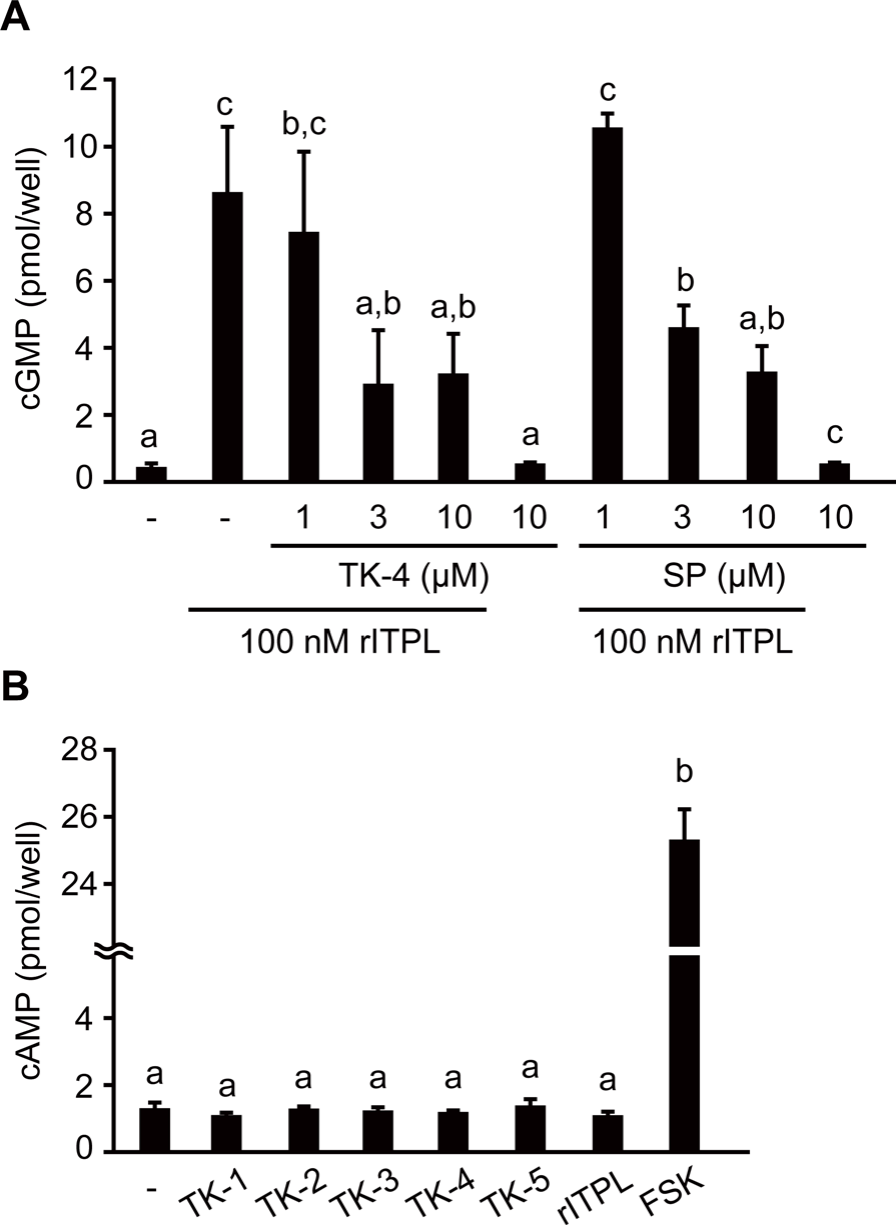
Effects of TK-4, rITPL, and spantide I on cyclic nucleotide levels in BmN cells. Intracellular cGMP (A) and cAMP (B) levels in BmN cells following 30-min incubation with the peptide and reagent of interest at the indicated concentrations were measured. (A) Effects of TK-4 and spantide I on the response of BmN cells to rITPL. The cGMP levels of the cells exposed to 100 nM rITPL with TK-4 or spantide I (SP) were determined. Responses to TK-4 alone, spantide I alone, rITPL alone, and vehicle treatment (-) were also examined. (B) The cAMP levels of the cells after incubation with 1 μM of five TRPs or rITPL. Responses to vehicle treatment (-) and 10 μM forskolin (FSK; positive control) were also examined. Data are presented as the mean + S.D. (*n* = 3 or 4). Different letters indicate significant differences (*P* < 0.05; Tukey’s HSD).

Previously, we showed that BNGR-A24 is expressed in *B*. *mori* ovary-derived BmN cells and that rITPL increased intracellular cGMP levels *via* BNGR-A24 [15]. The results of the current study show that 30-min exposure to 100 nM rITPL caused significant elevation of cGMP levels in the presence of the phosphodiesterase inhibitor IBMX (Fig 5A). In contrast, exposure to TK-4 at concentrations as high as 10 μM failed to increase cGMP levels (Fig 5A), suggesting that the effect of TK-4 on BNGR-A24 does not alter intracellular cGMP production in BmN cells. Since ITPL and TK-4 are competitive ligands of BNGR-A24, we hypothesized that TK-4 might inhibit the cGMP signal induced by ITPL *via* the binding of ITPL to BNGR-A24. To confirm this hypothesis, we added an excess of TK-4 to BmN cells exposed to rITPL and evaluated the stimulatory effect of ITPL on the cGMP signal. Addition of ≥3 μM TK-4 resulted in a significant decrease in rITPL-induced cGMP levels (Fig 5A), supporting our hypothesis. A similar result was obtained when spantide I was used as a potential competitor of ITPL. These findings suggest that the intracellular signaling pathways of TK-4 and ITPL *via* BNGR-A24 are different from each other, and that each of these ligands could inhibit the signaling cascade of the other through competitive binding to BNGR-A24. We also examined the effects of the peptides on intracellular levels of cAMP as a second messenger candidate responsible for the signaling pathways of TRPs and ITPL; 30-min exposure to 1 μM five TRPs or rITPL failed to stimulate cAMP production (Fig 5B). These results suggest that TRPs stimulate BNGR-A24 *via* different signaling pathway from cAMP and cGMP signaling at least on BmN cells.

## Discussion

Previously, we reported that BNGR-A24 is an ITPL receptor [15]. The present study showed that BNGR-A24, as well as BNGR-A32, are physiological receptors for endogenous TRPs. Our data also demonstrated the competitive binding of TRPs and ITPL to BNGR-A24, which may lead to ligand-specific signals.

### BNGR-A24 as a TRP receptor

Our results clearly indicate that BNGR-A24 is a functional receptor of five endogenous TRPs in *B*. *mori*, as predicted based on a prior report showing that heterologous DTK-6 activates BNGR-A24 [26]. The binding of TK-4 to BNGR-A24 was inhibited by the addition of spantide I. This result is consistent with previous findings that spantide I and its derivatives, spantide II and III, exhibit antagonistic effects against TRP receptors in *D*. *melanogaster* (*Drosophila* tachykinin receptor [DTKR] and neurokinin receptor from *Drosophila* [NKD]) [27,28] and a TRP receptor in the stable fly *Stomoxys calcitrans* [29]. A more recent study has shown that BNGR-A24 expressed in HEK293 and Sf21 cells responds to five *B*. *mori* TRPs, as well as to an additional TRP-like peptide (KPQFFVGVKa) derived from the same tachykinin precursor [30]. The authors found that TK-5 generates a weaker response than the other TRPs in the Ca^2+^ imaging assay in HEK293 cells; this is inconsistent with our data demonstrating that BNGR-A24 responds similarly to all five *B*. *mori* TRPs with nanomolar-order EC_50_s. Our results showing that of the five TRPs examined, BNGR-A24 exhibits the weakest response to TK-1 (Fig 2A and Table 1), are similar to those of the assay in Sf21 cells [30]. These differences in response may be due to variations in the assay systems; in particular, our heterologous expression system utilized co-expression of a promiscuous mouse Gα15 subunit, which couples with most GPCRs, and its consequent signaling *via* the increase in intracellular Ca^2+^ levels [31,32]. In this context, our results suggest that HEK293 cells do not possess an appropriate G protein alpha subunit for BNGR-A24 and that Gα15 might compensate for the activity of BNGR-A24 *via* TK-5 stimulation.

### Relationship between TRPs and natalisins in *B*. *mori*

Of the three putative TRP receptors in *B*. *mori*, BNGR-A24, an ortholog of DTKR [14,15,25], is functional as a receptor for all five TRPs. In contrast, the NKD orthologs, BNGR-A32 and BNGR-A33 [14,15,25], respond only to TK-5 or none of the TRPs, respectively. These results are consistent with previous reports of *D*. *melanogaster* TRPs and their receptors demonstrating inconsistencies between the response of DTKR to six endogenous TRPs [27] and the response of NKD to one TRP, DTK-6 [28]. A more recent study has shown that NKD-type GPCRs in various insect species, including BNGR-A32 and -A33, are characterized as natalisin receptors [26]. BNGR-A32 and -A33 have distinct preferences for ligands; the former receptor responds to natalisins with the C-terminal F*XXX*Ra motif, and the latter is a receptor for Y*XXX*Ra-containing natalisins. Three natalisins (NTL-1, -3, and -5) acting on BNGR-A32 with EC_50_ = 15–160 nM, share C-terminal F(F/W)(G/A)*X*Ra consensus sequences, which, except for the hydrophobic aromatic residue at the second position, are similar to those of TRPs. The second characteristic Phe residue is found in TK-5, but not in the other *B*. *mori* TRPs (Fig 1C). This may explain why BNGR-A32 responded only to TK-5 (Fig 2B). The natalisins of two other lepidopterans, *Danaus plexippus* (8/15) and *M*. *sexta* (12/15) also contain the F(F/W)(G/A)*X*Ra motif [26]. Since this motif is not found within natalisins in other insect orders [26], it may be a characteristic of ligands specific to lepidopteran natalisin/TRP receptors. Taken together, these findings indicate that BNGR-A32 is shared by TRPs and natalisins containing the FF*XX*Ra motif. Sharing of the same GPCR by neuropeptides of different families has been reported for RF*X*a-possessing peptides [33].

### Competitive action of ITPL and TRPs *via* BNGR-A24

Our finding that endogenous ITPL and TRPs bind to the same receptor was unexpected, since these peptides belong to two distinct peptide families with dissimilar amino acid sequences and structural characteristics. *B*. *mori* TRPs are linear polypeptides composed of 9–14 amino acids with a C-terminal pentapeptide consensus motif (Fig 1C), whereas *B*. *mori* ITPL is a 79-amino acid neuropeptide folded by three intramolecular disulfide bonds (Fig 1C), which is the characteristic of CHH family peptides [1–4]. The C-terminal region seems to be important for BNGR-A24 recognition of ITPL; *B*. *mori* ITP, which contains the same 40-amino acid N-terminus as ITPL [34], exhibits much lower affinity to BNGR-A24 than ITPL [15]. However, unlike ITP and most CHH family peptides that are C-terminally amidated, the C-terminal sequence of ITPL is not subject to amidation and differs from the consensus C-terminal motif found in TRPs. Therefore, the binding mechanism of ITPL to BNGR-A24 is dissimilar to that of the TRPs. Since our results indicate competitive binding of ITPL and TRPs to BNGR-A24, the regions on the receptor responsible for interacting with these two ligands must be sufficiently proximal to affect the binding of each ligand.

The response of BNGR-A24 to TRPs was 3.7–96-fold higher than to ITPL, with EC_50_ = 26 nM [15]. Consistent with the signaling of other TRPs [20], it has shown that *B*. *mori* TRP signaling *via* BNGR-A24 is mediated by cAMP and Ca^2+^, concomitant with the recruitment of two G protein alpha subunits, Gs and Gq, in heterologous HEK293 and Sf21 cells [30]. However, the present study showed that none of endogenous *B*. *mori* TRPs induced intracellular cAMP production in BmN cells (Fig 5B), suggesting that different signaling pathways such as Ca^+^ signaling are responsible for TRPs function at least on BmN cells. We also demonstrated that ITPL failed to increase cAMP levels in BmN cells (Fig 5B); these results are inconsistent with a previous report showing that ITP plays a role in ion fluid modulation in the locust ileum *via* both cAMP and cGMP [35], although ITPL has not been examined. These results indicate that ITPL is not a partial agonist of TRPs, but rather an agonist acting through a distinct signaling pathway. Our data also showed that TRP failed to increase cGMP levels in BmN cells and that ITPL-activated cGMP production was inhibited by TK-4 and spantide I (Fig 5A). The presence of excess TK-4 inhibited the interaction of ITPL with BNGR-A24 and *vice versa* (Fig 4), indicating that these peptides act as ‘orthosteric ligands’ rather than ‘allosteric modulators’ [36,37].

As discussed above, our results suggest that TRP and ITPL signaling are independent; thus, these two families of neuropeptides may exert distinct functions *via* the same receptor. Such ‘ligand-biased signaling’ is an accepted concept, according to which multiple orthosteric ligands have the ability to bias their signaling between different G proteins and/or between G proteins and β-arrestins, contributing to stabilization of the receptor in a ligand-specific conformation [37]. Such a ligand signaling selectivity has been observed for the TRP receptor in *S*. *calcitrans* when the receptor was expressed in heterologous S2 cells; substitution of Ala by Gly within the C-terminal F*X*G/A*X*Ra motif of an endogenous TRP resulted in full agonistic activity against Ca^2+^ signaling, whereas cAMP was not affected [38]. Another study has shown the significance of β-arrestin signaling in the anti-apoptotic effects exerted by substance P *via* the neurokinin 1 receptor [39], suggesting that tachykinin receptors may function *via* β-arrestin signaling in addition to G protein-mediated signaling. Although the functional consequences of β -arrestin-mediated signaling have yet to be determined, the vertebrate tachykinin receptors, *Drosophila* DTKR and NKD, have been shown to recruit β-arrestin 2 in a heterologous expression system following activation by an endogenous ligand [40]. Since it remains unclear whether the ITPL signaling effector couples directly with BNGR-A24 leading to activation of guanylyl cyclases, it is important to determine which signaling pathway is facilitated by ITPL following its binding to BNGR-A24.

The fact that ITPL and TRPs act competitively on the same receptor also points to a functional relationship. Previous studies have suggested feeding-stimulatory effects of *B*. *mori* TK-1 and TK-2 [24] and the participation of BNGR-A24 in feeding [30]; thus, TRP and ITPL signaling may coordinate to regulate feeding behavior *via* BNGR-A24. Our previous findings indicate that starvation may potentiate ITPL signaling by up-regulating both the ligand and its receptor in intestinal tissues [15]. The present finding that *tk* expression levels were unchanged in intestinal tissues following 24-h starvation (Fig 1A), suggests that locally expressed TRPs in these tissues may not be involved in regulation of the ITPL signaling, at least at the transcriptional level.

In conclusion, our present study demonstrates that endogenous TRPs and ITPL act competitively on the same receptor, BNGR-A24. In addition, BNGR-A32 functions as a physiological receptor for TK-5 and natalisins containing the C-terminal F(F/W)(G/A)*X*Ra consensus sequence. These findings provide an important clue to understanding the biological functions of ITPL, TRPs, and natalisins. Our data suggest the ligand-biased signaling systems *via* BNGR-A24, although the downstream effectors of TRPs and ITPL remain to be elucidated. Since the occurrence of such ligand-biased signaling systems involving endogenous ligands of different peptide families is rare, these GPCRs constitute interesting models for understanding how complicated signaling systems contribute to coordinated regulation. Our present data also further our understanding of ligand-receptor co-evolution; tachykinin family peptides, including TRPs, are found in vertebrates and invertebrates, while CHH family peptides, including ITPL, are unique to ecdysozoan species. Since BNGR-A24 is the only TRP receptor-like GPCR identified as an ITPL receptor, the next crucial step would be to determine whether the competitive binding of TRPs and CHH family peptides is a common phenomenon in ecdysozoan species.
